# A Rare Genetic Mutation Leading to a Deficiency of Adenosine Deaminase 2 Enzyme in a Long-Standing Case of Cutaneous Polyarteritis Nodosa: A Case Report

**DOI:** 10.7759/cureus.30295

**Published:** 2022-10-14

**Authors:** Rutu A Contractor, Yash D Bhavsar, Arpit P Joshi, Niyati N Pujara, Dhaiwat M Shukla

**Affiliations:** 1 Medical Student, Smt. Nathiba Hargovandas Lakhmichand Municipal Medical College, Ahmedabad, IND; 2 Internal Medicine, Vadilal Sarabhai General Hospital, Ahmedabad, IND; 3 Medical Student, Odessa National Medical University, Odessa, UKR; 4 Internal Medicine, Byramjee Jeejabhoy Medical College, Ahmedabad, IND; 5 Internal Medicine, Kursk State Medical University, Kursk, RUS; 6 Rheumatology, Vadilal Sarabhai General Hospital, Ahmedabad, IND; 7 Rheumatology, Narayana Multispeciality Hospital, Ahmedabad, IND

**Keywords:** foot ulcer, chronic inflammatory demyelinating polyneuropathy (cidp), dada2, cecr1, genetic syndromes, cutaneous polyarteritis nodosa

## Abstract

Vasculitis is an inflammatory disorder of blood vessels affecting multiple organs. A deficiency of adenosine deaminase enzyme type 2 (DADA2) is a novel condition identified as a monogenic cause of cutaneous vasculitis. Since its first description in 2014, numerous case series and literature across several countries have expanded the scope of our understanding of this rare genetic condition. However, due to a scarcity of reported cases in adults, little is known regarding its full spectrum of clinical presentations, treatment guidelines, or outcomes in the adult patient population. It is established that it commonly affects multiple organ systems, such as the skin, musculoskeletal, neurological, hematological, gastrointestinal, and renal systems. It presents with a wide range of clinical manifestations, including fever, Livedoid rash, cutaneous polyarteritis nodosa, polyneuropathy, and immunodeficiency. Such a varied clinical spectrum opens an opportunity for discussion to list some of the differential signs of DADA2. In this article, we report a unique case of a 26-year-old male with a delay of nine years in diagnosing a genetic mutation that led to DADA2. In addition, a 10-year history of recurring cutaneous ulcers and peripheral neuropathy makes this case a noteworthy addition to the literature on cutaneous vasculitis and its miscellaneous causes.

## Introduction

First described in 1866 by Adolph Kussmaul and Rudolph Maier, polyarteritis nodosa (PAN) is a systemic necrotizing vasculitis. The limited version of the disease is known as cutaneous polyarteritis nodosa (CPAN). Even with the limited form, there is significant morbidity secondary to digital ulcerations, ischemia, and painful skin nodules. Patients with CPAN can progress to systemic polyarteritis nodosa (SPAN); however, it is a rare occurrence. In addition to hepatitis B, hepatitis C, and malignancies such as hairy cell leukemia, the secondary causes of PAN have recently been explored extensively. Biallelic loss of function mutations in the *cat eye syndrome critical region protein 1* (*CECR1*)/*adenosine deaminase 2* (*ADA2*) gene leads to a deficiency of the ADA2 protein. The encoded protein is one of two adenosine deaminases found in humans that regulate the levels of signaling molecules. The encoded protein is secreted from monocytes and may regulate cell proliferation and differentiation. This gene may be responsible for some of the phenotypic features associated with cat eye syndrome and has been implied as a novel cause of CPAN since 2014. It follows an autosomal recessive mode of inheritance [1,2]. Appearing like an isozyme chemically, the ADA2 enzyme is distinct in structure and function from ADA1. ADA1 is present in several human tissues, although its level is comparatively lower in plasma. It plays a catalytic role in the purine degradation pathway for the deamination of adenosine and deoxyadenosine nucleotides. On the other hand, ADA2 plays the role of a growth factor in immune cell differentiation and proliferation. ADA2 activity is profoundly elevated in plasma from patients suffering from liver diseases, such as chronic hepatitis and cirrhosis, acquired immunodeficiency syndrome, adult T-cell leukemia, acute lymphoblastic leukemia, tuberculosis, and diabetes mellitus [3-5]. However, it is mystifying to know that ADA2 has a higher-order Michaelis constant (Km) than ADA1, for which several theories have been suggested [6-8]. ADA1 deficiency causes T-cell dysfunction by intracellular accumulation of toxic compounds that cannot be removed, leading to a diagnosis of severe combined immunodeficiency syndrome (SCID) in young children, whereas ADA2 deficiency presents as a much milder phenotype, with a clinically distinct case presentation, as elaborated further in this case report [8]. Although the majority of ADA2 deficiency cases described in the literature present with vasculopathy, cases with defects exclusively in the hematopoietic system such as pure red cell aplasia (PRCA) or Diamond-Blackfan anemia have also been reported [9].

ADA2 is predominantly present in myeloid cells and monocytes and plays a role in the T-cell-dependent differentiation of monocytes to macrophages [10]. It acts as a growth factor for endothelial cells and the maturation of M2-type macrophages because of which the deficiency of ADA2 leads to increased production of proinflammatory M1 macrophages, resulting in a vasculitis-like disease [8]. Another novel mechanism reported to play a role in its pathogenesis is the formation of neutrophil extracellular traps (NETs) in the absence of ADA2 which plays a role in macrophage differentiation and production of excess tumor necrosis factor-alpha (TNF-α) [11,12]. This mechanism may explain the efficacy of anti-TNF-α medications in the treatment of deficiency of adenosine deaminase enzyme type 2 (DADA2). Over the years, a few mutations have been identified for causing ADA2 deficiency, including missense, nonsense, genomic deletions, and splicing mutations [8]. Even among individuals who are homozygous for the same mutations, there are large differences in their individual phenotypes. Hence, epigenetic modifiers and environmental factors should also be considered while eliciting the causes and manifestations of ADA2 deficiency [13].

Initially reported as a case series of 34 patients by two different groups, more than 300 cases have been reported worldwide [1,2,14]. However, DADA2 remains an underdiagnosed condition due to its strong phenotypic variability (fever, myalgia, rash, early onset of strokes, and hematological, neurological, and intestinal manifestations) which generates a difficult question about knowing the perfect time to raise a clinical suspicion [1,2]. Such a diagnostic dilemma is to blame for a delay in the diagnosis and treatment of affected patients who remain at an elevated risk of morbidity and mortality. The clinical presentation in patients with DADA2 is diverse. The age of presentation is variable; however, among the cases reported till now, more than half have been reported in children less than five years of age [15]. Longitudinal cohort studies in patients have shown that the disease manifestations can be divided into three major phenotypes, namely, inflammatory/vascular, immune dysregulatory, and hematologic; however, most patients present with a combination of these three phenotypic groups. The cardinal features of the inflammatory/vascular group included cutaneous manifestations resembling CPAN and cerebrovascular stroke which explains the signs and symptoms of our patient presenting with recurrent skin ulcerations and peripheral neurologic symptoms such as tingling and numbness in the distal extremities.

To date, diagnostic criteria for confirmation of DADA2 are not clearly established in the literature. However, the first meaningful step is to check the plasma activity of ADA2, with the help of screening tests such as enzyme-linked immunosorbent assay (ELISA) that detects low levels of ADA2 activity in symptomatic cases, as described in the literature [16,17]. The screening test should be followed by confirmatory genetic testing to look for specific target mutations. The standard methods in genetic testing currently in use include Sanger sequencing, next-generation sequencing, and exome sequencing. Our case was identified by Sanger sequencing which confirmed pathogenic homozygous mutation at exon 2. Even after appropriate validation of these tests, a smaller group of mutations could be missed. Techniques such as multiplex ligation-dependent probe amplification (MLPA) and long-range polymerase chain reaction (PCR) may be considered as alternative or second-line diagnostic tests to assist in establishing a diagnosis [18].

## Case presentation

**Figure 1:**
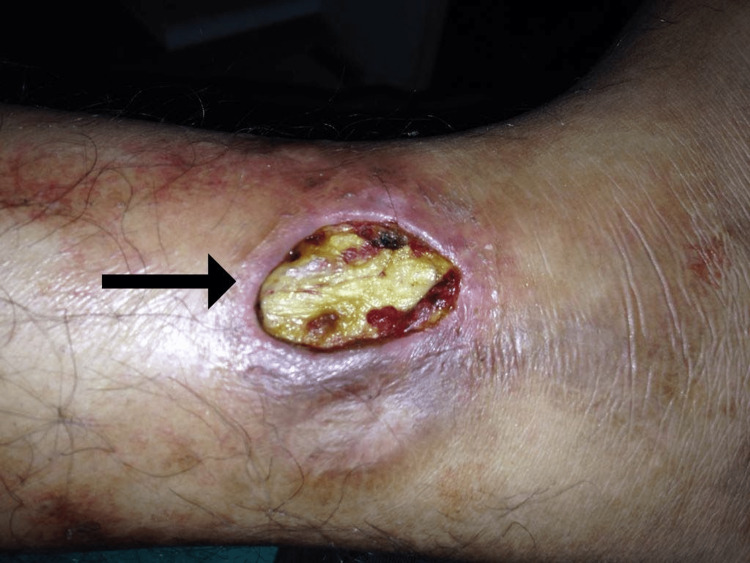
A deep skin ulcer measuring 4 cm × 2 cm located 1 cm above the left medial malleolus. The ulcer was non-gradable due to the presence of a yellowish slough completely covering the base of the ulcer.

A 26-year-old male belonging to the Agarwal community presented to the hospital at the age of 21 years with the chief complaint of a painful and chronic deep skin ulcer on the left lower limb along with fever. The ulcer measured 4 cm × 2 cm in size and was located 1 cm above the left medial malleolus (Figure 1). He complained of recurring smaller-looking ulcers over the left lower limb from 2012 to 2016. On physical examination, the edges of the ulcer were found to be undermined along with the presence of erythema. The base was covered in a yellowish-appearing slough making it difficult to grade the ulcer appropriately. He had a medical history of bilateral ankle pain at the age of seven (treated symptomatically with non-steroidal anti-inflammatory drugs and serratiopeptidase), which eventually progressed to involve both knee joints later in his life. During that period, he also had an episode of afebrile generalized tonic-clonic seizure for which he was treated in-patient with diazepam and started on a maintenance dose of oral carbamazepine for two years. It was later stopped as he continued to remain seizure-free on a tapering dose of the antiepileptic drug. Electroencephalography at that time showed signs of epileptiform activity in the left frontal lobe, but magnetic resonance imaging revealed no intracranial abnormalities simultaneously. His family history was significant for parapsoriasis in his mother. He reported no significant allergic reactions to any food or medications and denied using any illicit substances.

**Figure 2:**
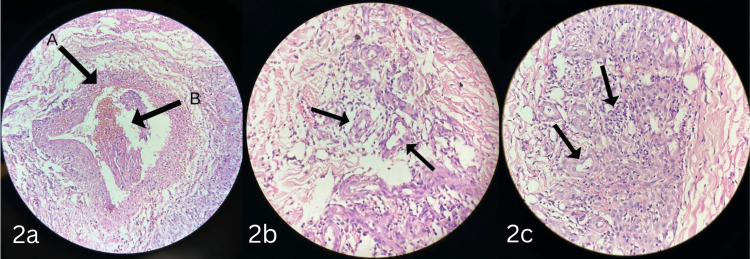
Histopathology slides of the ulcer biopsy with hematoxylin and eosin staining. (a) (40× magnification) (A) vessel wall thickening and fibrosis, and (B) thrombus occluding the lumen. (b) (20× magnification) small-vessel vasculitis consistent with inflammatory infiltrates surrounding them. (c) (20× magnification) shows the interstitial accumulation of neutrophils and eosinophils along with fibrosis.

**Figure 3:**
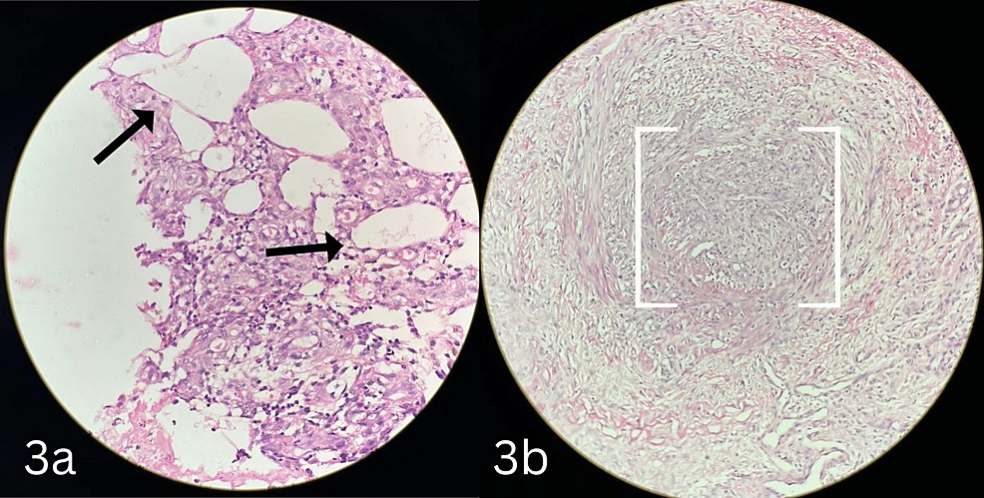
Histopathology slides of the ulcer biopsy with hematoxylin and eosin staining. (a) (10× magnification) panniculitis (subcutaneous fat inflammation) that is commonly seen with cutaneous polyarteritis nodosa. (b) (4× scanner view) blood vessels filled with neutrophilic infiltrates and recanalization consistent with healed vasculitis.

A biopsy of the skin lesion in the left lower leg was collected and sent for histopathology. It showed intimal vessel proliferation and thickening seen along with thrombosis (Figures 2a, 2b) leading to Ischemia and ulceration. In addition, it showed an infiltrate mixed with neutrophils and eosinophils in and around the vessels involving the panniculus causing panniculitis (Figure 3a) and blood vessels filled with neutrophilic infiltrate and recanalization consistent with healed vasculitis (Figure 3b).

Given the pathological findings, additional blood work to assess an underlying vasculitis was obtained. The antinuclear antibody screen was negative, and antineutrophil cytoplasmic antibody (ANCA) indirect immunofluorescence was negative for both perinuclear ANCA and cytoplasmic ANCA. The patient tested negative for hepatitis B and hepatitis C, and the liver function tests were normal. Human immunodeficiency virus (HIV) infection was also ruled out by a negative fourth-generation HIV antigen/antibody testing. These findings pointed toward a diagnosis of CPAN. He was advised to start treatment with oral prednisolone, colchicine, and prophylactic antibiotics in mid-2012. In 2013, he presented with a new-onset tingling, pain, and weakness along the median nerve distribution of the left and right upper extremity which was later diagnosed as mononeuritis multiplex associated with the vasculitis. He underwent a series of diagnostic tests over the course of his illness to elicit an underlying pathology. Arterial Doppler ultrasonography of the left lower limb showed a pattern of monophasic flow in the left tibial and dorsalis pedis arteries suggesting peripheral artery disease. The electromyography and nerve conduction studies (NCS) were performed which showed segmental demyelination with axonal motor and sensory polyneuropathy involving all four limbs. NCS findings of the left median nerve are shown in Table 1.

**Table 1:** Nerve conduction study findings. Nerve conduction study showed prolonged distal motor latency in the left median nerve with reduced motor nerve conduction velocity in the forearm. Compound muscle action potential was >50% reduced in the left median nerve proximal to the wrist along with evidence of temporal dispersion. Distal sensory latency was marginally prolonged, bilaterally, in median nerves.

Site	Latency	Duration	Amplitude	Area	Segment	Distance	Interval	Nerve conduction velocity
Left median nerve								
Wrist	5.4 ms	7.7 ms	6.5 mV	15.3 mVms	Wrist	Not reported	5.4 ms	Not reported
Elbow	11.7 ms	11.8 ms	3.0 mV	8.5 mVms	Wrist-elbow	250 mm	6.3 ms	39.7 m/s

MRI of the spine showed a posterior bulge of C3-C4 and L5-S1 intervertebral discs causing indentation over the ventral aspect of the dural theca but did not show any evidence of significant extradural compression or canal stenosis. Cardiac pathologies were ruled out by transthoracic echocardiography and computed tomography angiography of the chest and abdomen. In 2014, he was started on an all-oral regimen of azathioprine and corticosteroids, which temporarily improved his symptoms; however, the condition continued to recur. Due to his unremitting course of autoimmune symptoms, mycophenolate mofetil was instituted with an initial dose of 500 mg PO BID which was later titrated up to 1,500 mg PO BID. Unfortunately, despite receiving maximum tolerable doses, the symptoms continued to recur until 2016. Over the next few months, the efficacy of tacrolimus (calcineurin inhibitor) was tested for this patient. However, it was later discontinued due to a lack of improvement in his symptoms. As a last resort, the use of monoclonal antibodies against CD20 (e.g., rituximab) was considered to manage his condition and was discussed with the patient. After taking appropriate consent, he was scheduled to receive two doses of 1 g rituximab 15 days apart which resulted in sustained remission of the ulcer and bilateral upper extremity mononeuritis multiplex from 2017 to 2021. During the same time, methotrexate and folic acid were prescribed at a weekly maintenance dose as part of a combined medication regimen. However, a long-term course of immunosuppressants led to multiple systemic complications such as acute gastroenteritis, an episode of shingles caused by herpes zoster virus, and recurrent urinary tract infections with a frequency of about three to four episodes a year. Immunocytology reports showed reduced CD19 cell count and immunoglobulin (Ig)G and IgM levels which could be either attributed to a chronic course of immunosuppressant medications or due to the inherent deficiency of ADA2. A summary of the symptoms occurring in the patient over time is described in the flowchart below.

**Figure 4:**
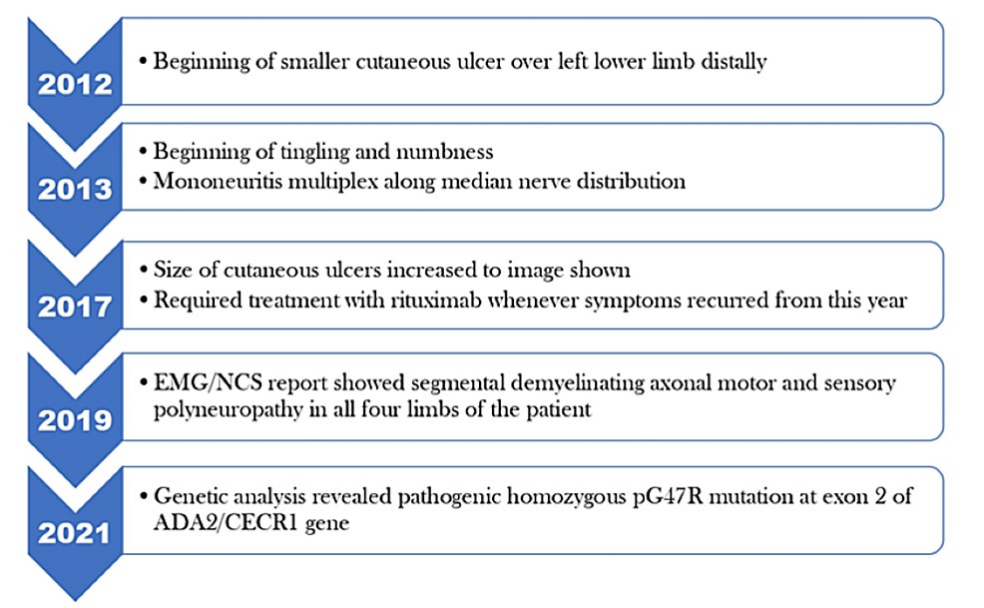
Flowchart describing the series of events and symptoms seen in the patient over time. EMG: electromyography; NCS: nerve conduction study

After an unrelenting trial of various immunosuppressants with limited effectiveness, he was advised to send his blood sample to the Hematology and Oncology Unit of the Postgraduate Institute of Medical Education and Research, Chandigarh, in April 2021. Genetic analysis by Sanger sequencing of exon 2 to exon 10 was performed which was positive for a pathogenic homozygous pGly47Arg mutation at exon 2 in the *ADA2*/*CECR1* gene. The patient was diagnosed with DADA2 nine years after his initial presentation. He is currently undergoing treatment with adalimumab (40 mg subcutaneously) every 15 days. This therapeutic option acts by inhibiting TNF-α. Except for mild anemia of chronic disease, the current laboratory findings of the patient are unremarkable.

## Discussion

This case report describes a patient suffering from a chronic course of recurrent CPAN who presented with a longstanding history of deep cutaneous ulcers and peripheral neuropathy. In our case, symptoms and presentations were identified to be linked with the effects of reduced ADA2 enzyme activity. Vasculitis of such a rare form is characterized by varied age of onset, severity, and organ involvement, even within families and among patients with the same mutations. Manifestations range from severe or fatal systemic vasculitis or multiple strokes in children to limited cutaneous manifestations in middle-aged people. While this entity presents chiefly in the pediatric age group (less than 10 years of age), the first clinical symptom in our case manifested at the age of 16, and after a nine-year delay, a definitive diagnosis of DADA2 mutation was established. The clinical picture of DADA2 appears to be closely related to the age of onset. An increased incidence of the vascular phenotype is found in adult-onset DADA2, while childhood cases present more commonly with hematological phenotypes that can be potentially treated with hematopoietic stem cell transplant (HSCT). However, genotype-phenotype correlations in DADA2 have been difficult to establish because of incomplete penetrance and variable clinical manifestations in patients with identical mutations [12]. Cases reported in academic literature have shown a nearly identical incidence in male and female populations, with close to 53% of confirmed cases being male [8]. The patient in our case report belonged to the Agarwal community of India and had a p.Gly47Arg mutation. In two of the published case series on DADA2 patients in India, the most common mutation was p.Gly47Arg, and most people having this mutation did, in fact, belong to the Agarwal community, mirroring our case presentation [19,20].

DADA2 often manifests in adults with multisystem involvement, but small and medium-vessel vasculitis, in particular, presents among more than three-fourths of the cases documented. This creates a significant overlap among patients presenting with a regular case of CPAN. However, as reported in the literature, features of the central nervous system (CNS), gastrointestinal, and cardiac involvement are more suggestive of the diagnosis of DADA2 vasculitis over CPAN. The incidence of stroke is reported more frequently in DADA2 cases, while the peripheral nervous system involvement causing mononeuritis multiplex is more commonly reported in CPAN. In our case, a diagnosis of mononeuritis multiplex and segmental demyelinating polyneuropathy was recorded after the patient presented with tingling and numbness in the upper extremities. After a thorough neurological examination, no underlying abnormal signs of CNS involvement were noted, except for an episode of generalized tonic-clonic seizure in childhood. Although skin involvement is usually more common among patients with PAN, for patients presenting with DADA2, lesions such as livedoid rash, ulcerations, maculopapular rash, petechial rash, and subcutaneous nodules are also not uncommon. Our case presented with a history of recurring ulcers for a long time before the diagnosis of DADA2 mutation was established. The incidence of constitutional symptoms such as fever and myalgias is increasingly found in systemic PAN compared to DADA2. We did not report any such characteristic constitutional symptoms in our case to date. Due to the diagnostic dilemma and DADA2 being a novel entity, our patient had to face multiple diagnostic challenges. One of the most important factors contributing to a sluggish response in drafting an appropriate management plan for this patient was a lack of resources and expertise in the field. The sample had to be sent from Ahmedabad to Chandigarh which took over 15-20 days to return. For this reason, it is important to consider the practical challenges associated with the availability, cost, and affordability of such novel genetic tests, especially in resource-deficient facilities in developing countries like India. A case series among Indian patients found the median gap between onset and diagnosis to be 52 months [19].

Immunosuppressive therapy has been the mainstay of treating DADA2-related vasculitis and stroke; however, it is only partially successful and may prove to be unreliable in treating the underlying core hematological defects. Steroids (prednisolone or dexamethasone) were most commonly used in the past, although their use was limited due to recurring inflammatory and vasculitis episodes upon tapering the medication. With limited success, other immunomodulators such as azathioprine, cyclosporine, tacrolimus, cyclophosphamide, and methotrexate have also been used in the past. Tocilizumab (an interleukin-6 inhibitor) effectively controlled inflammation in select DADA2 patients having Castleman-like presentation but failed to adequately control or prevent recurrent stroke episodes in DADA2 patients. At present, the mainstay of treatment consists of biologic agents such as anti-TNF-α medications (e.g., etanercept, infliximab, and adalimumab) which prove to be reliable at controlling the fever episodes, vasculopathy, and the number and lethality of stroke episodes in all patients reported across the DADA2 spectrum. However, TNF inhibitors should be used cautiously considering their potential for inducing serious side effects such as reactivation of latent infections and allergic reactions. HSCT is being increasingly studied as a potential definitive treatment for DADA2 patients. A cohort study of 14 patients from six countries who received HSCT for DADA2 was reported by Hashem et al. [9]. All 14 patients in the study were cured of their disease and its manifestations, including vasculopathy, with no significant post-transplant complications [9]. Unlike the standard of care for stroke patients, it is not recommended to start acetylsalicylic acid and other anticoagulants as the risk of developing a hemorrhagic stroke is statistically higher in DADA2 patients [8]. We hope that our case report can prove to be a valuable addition to the literature on stroke, PAN, and DADA2. We intend to support the medical community globally in creating comprehensive potential diagnostic and management approaches for DADA2.

## Conclusions

We report a rare case with the intention to provide a systematic and multifaceted approach while analyzing the possible differential diagnosis for conditions that involve recurring skin ulcers and vasculitis. This can likely guide physicians in correctly identifying and exploring the underlying pathology for the secondary causes of CPAN. It can also provide more clarity in constructing an appropriate and timely management plan for patients suspected to have this condition, which, unfortunately, was not possible in our case due to a lack of resources. Since first described in 2014, DADA2 publications have evolved considerably over time, adding a diverse range of clinical presentations under its umbrella. Hence, expanding our understanding and changing our outlook in correlating the symptoms which were initially thought to be purely due to CPAN becomes inevitable. Special attention should be given to cases resembling CPAN, with a positive family history of skin ulcers, a personal history of stroke, peripheral neuropathy, or treatment resistance to two or more immunosuppressant medications prescribed for CPAN. In our case, the patient had a poor initial response to the standard treatment regimen for PAN, which narrowed the differential diagnosis toward ADA2 deficiency. Treatment options are yet to be defined clearly in the literature for DADA2 but current reports suggest anti-TNF-α medications as a standard of longitudinal care for all age groups. Positive results of HSCT show potential for cure in the future, especially when administered at an earlier age. However, it requires extensive large-scale studies to prove its true efficacy.
